# Superantigens in Cancer Immunotherapy: Mechanisms, Engineering Strategies, and Therapeutic Potential

**DOI:** 10.1096/fj.202602532R

**Published:** 2026-08-31

**Authors:** Urooj Yousaf Virk, Hafiza Aasia Malik, Muneera Anwer, Jennifer Wilson, Md. Sifat Rahi, Ming Q. Wei

**Affiliations:** ^1^ School of Pharmacy and Medical Sciences Griffith University Southport Queensland Australia

**Keywords:** antibody‐superantigen fusion, cancer immunotherapy, immune‐redirecting therapies, superantigens, T‐cell activation, tumor microenvironment, tumor‐targeted superantigens

## Abstract

Cancer immunotherapy has reshaped modern oncology by enabling the immune system to recognize and eliminate malignant cells. However, many solid tumors still respond poorly because of weak T‐cell activation and an immunosuppressive tumor microenvironment. Bacterial superantigens (SAgs) represent a unique class of immunomodulatory proteins capable of overcoming these limitations through direct activation of large T‐cell populations. Unlike conventional antigens, superantigens bypass classical antigen processing by simultaneously binding major histocompatibility complex class II molecules and T‐cell receptor Vβ domains, triggering rapid cytokine release and extensive immune activation. Although this potent mechanism has historically been associated with severe systemic toxicity, recent advances in protein engineering and targeted delivery have renewed interest in their therapeutic potential. This review discusses the structural and immunological basis of superantigen activity and highlights emerging strategies designed to improve tumor specificity and safety, including engineered low‐toxicity variants, antibody‐superantigen fusion proteins, nanoparticle‐based delivery systems, and tumor‐targeted constructs. We further examine how superantigens reshape the tumor microenvironment and synergize with immune checkpoint blockade, adoptive cell therapies, and other T‐cell‐redirecting approaches. Together, these advances position engineered superantigens as promising immune‐amplifying platforms with the potential to complement existing cancer immunotherapies and improve responses in poorly immunogenic tumors.

## Graphical abstract

Schematic overview of superantigen‐mediated antitumor immune responses and immune suppression within the tumor microenvironment. (1) Antigen‐presenting cells activate T cells through MHC‐II/TCR interactions resulting in the production of cytokines (2) The pro‐inflammatory cytokines released by activated immune cells stimulate downstream immune responses and promote differentiation of T‐helper cell subsets. (3) Effector T cells mediate direct tumor cell killing through cytokine secretion and apoptosis‐inducing mechanisms. (4) Within the TME, regulatory T cells, myeloid‐derived suppressor cells, and M2 macrophages promote immune evasion and dampen antitumor activity. (5) Immune checkpoint blockade targeting PD‐1/PD‐L1 restores T‐cell function and enhances antitumor cytotoxicity. Together, these mechanisms highlight the interplay between immune activation and tumor‐induced immunosuppression in cancer immunotherapy.

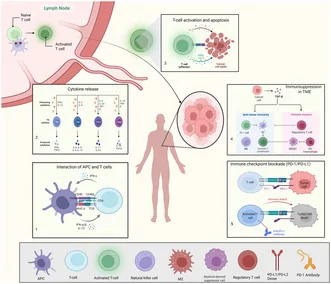

## 1 Introduction

**FIGURE 1:**
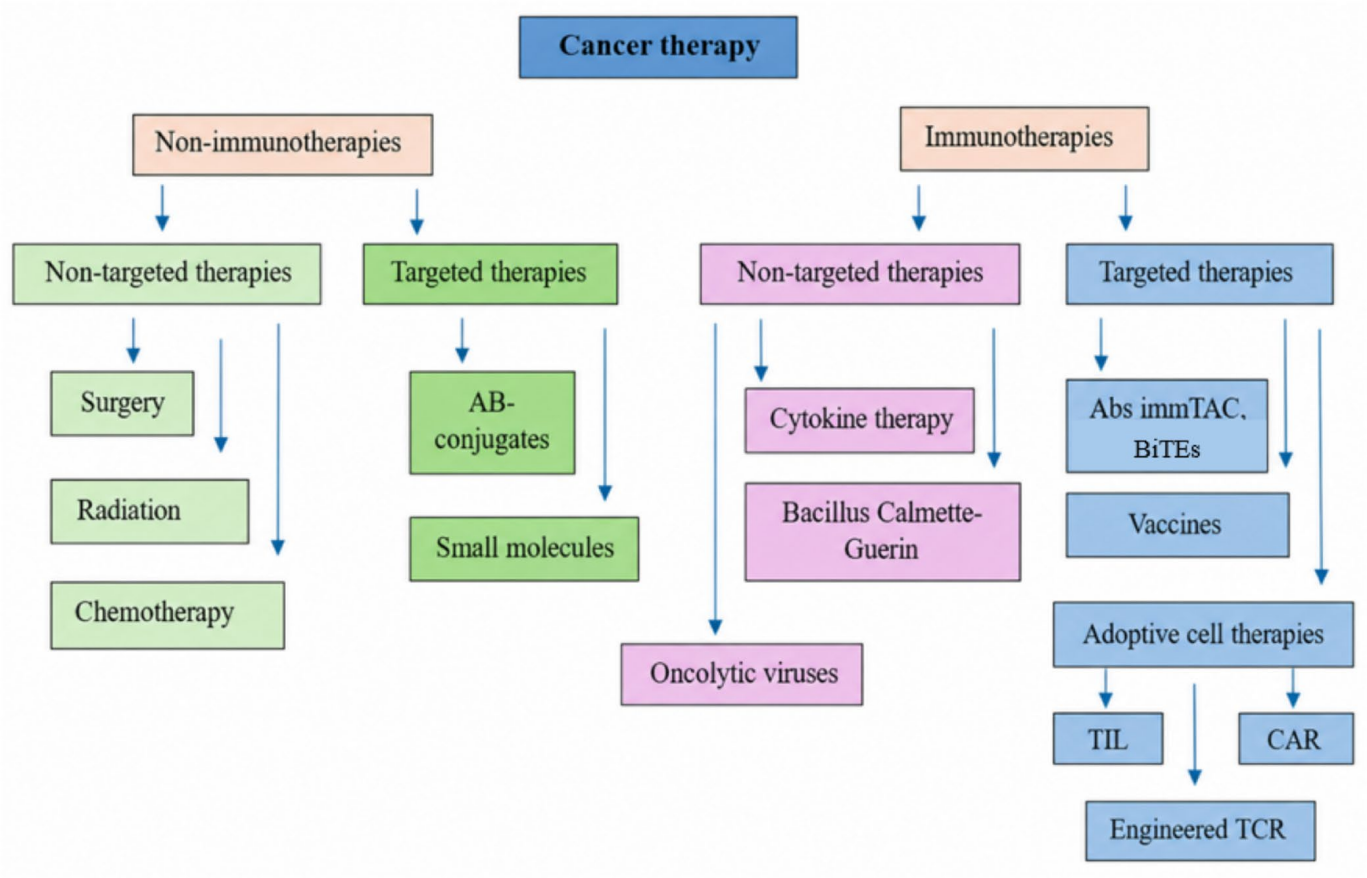
Classification of various cancer therapies based on their mechanisms of action. Cancer therapies are broadly divided into non‐immunotherapies and immunotherapies, each further categorized into nontargeted and targeted therapies. Non‐immunotherapies include surgery, radiation, chemotherapy, antibody‐drug conjugates (AB‐conjugates), small molecules, and oncolytic viruses. Immunotherapies consist of nontargeted approaches like cytokine therapy and Bacillus Calmette–Guérin (BCG), and targeted approaches including monoclonal antibodies (Abs), immune mobilizing monoclonal T‐cell receptors against cancer (immTACs), bispecific T‐cell engagers (BiTEs), cancer vaccines, and adoptive cell therapies such as tumor‐infiltrating lymphocytes (TILs), chimeric antigen receptor (CAR) T cells, and engineered T‐cell receptors (TCRs).

**FIGURE 2:**
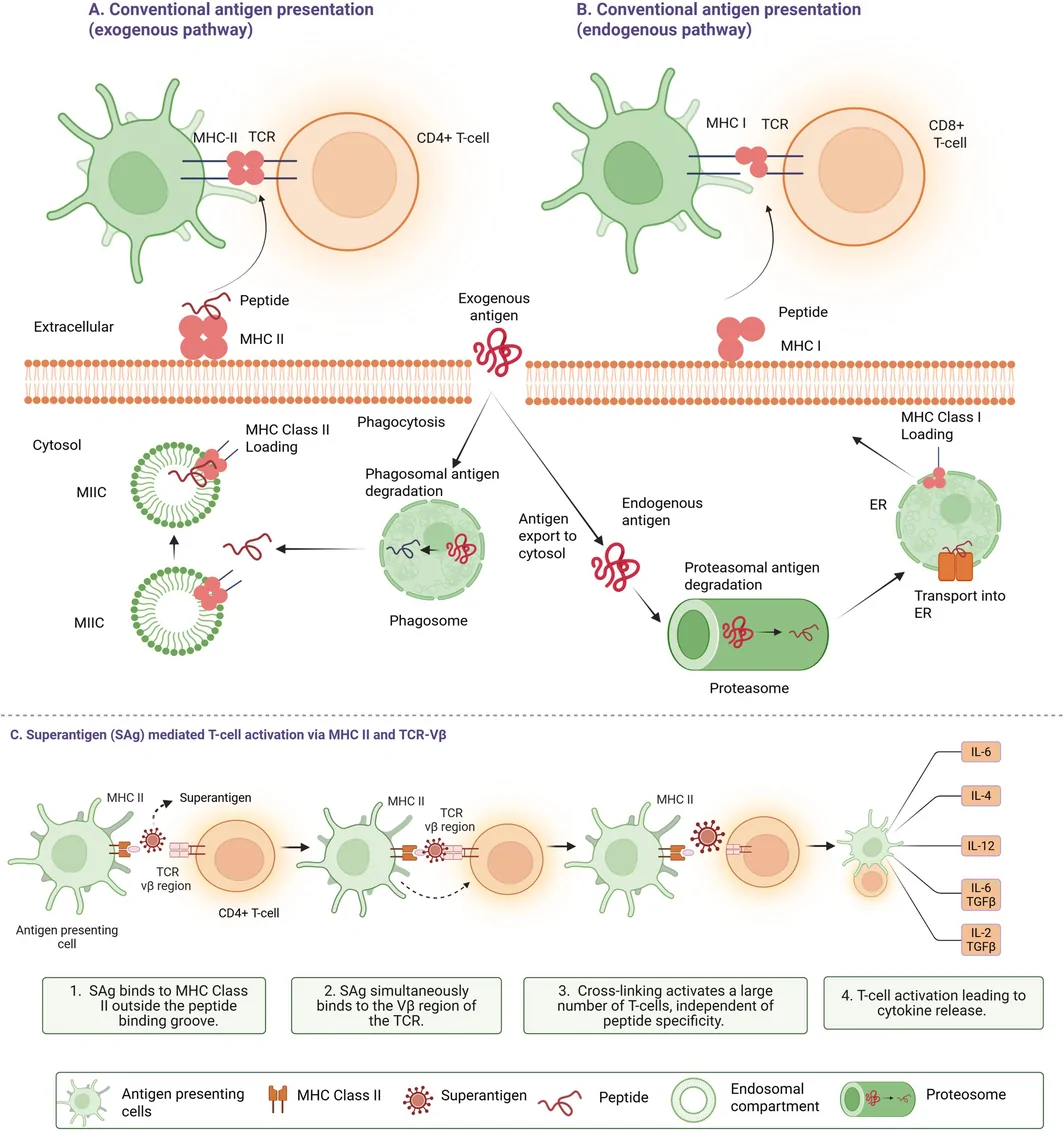
Antigen processing and presentation pathways via MHC class I and MHC class II molecules. Illustration shows how endogenous and exogenous antigens are processed and presented to T cells. Endogenous antigens (such as viral or tumor‐derived proteins) are degraded by the proteasome in the cytosol, and resulting peptides are transported into the endoplasmic reticulum (ER), where they are loaded onto MHC class I molecules. These MHC I–peptide complexes are then presented on the cell surface to CD8^+^ cytotoxic T cells. Exogenous antigens (such as extracellular pathogens) are internalized via phagocytosis, degraded within phagosomes, and loaded onto MHC class II molecules within specialized vesicles known as MIIC compartments. The MHC II–peptide complexes are then expressed on the cell surface and recognized by CD4^+^ helper T cells. Together, these pathways enable the immune system to detect and respond to both intracellular and extracellular pathogens.

Cancer remains one of the leading causes of mortality worldwide, accounting for nearly one in six deaths globally [1]. Despite advances in conventional treatment strategies such as surgery, chemotherapy, and radiotherapy, many malignancies remain difficult to treat because of therapy resistance, tumor heterogeneity, and treatment‐associated systemic toxicity (Figure 1); [2]. Over the past two decades, immunotherapy has emerged as a transformative approach in oncology by harnessing the immune system to recognize and eliminate malignant cells. Strategies including immune checkpoint blockade, adoptive T‐cell therapy, and bispecific antibodies have demonstrated that durable tumor regression can be achieved when immune effector cells are effectively redirected toward tumor tissue [3, 4].

Despite these advances, the clinical success of immunotherapy remains uneven across tumor types, particularly in solid tumors. A major obstacle is the immunosuppressive tumor microenvironment (TME), which can limit effective immune activation and prevent durable antitumor responses. Many tumors exhibit reduced T‐cell infiltration, impaired antigen presentation, and enrichment of suppressive immune populations such as regulatory T cells and myeloid‐derived suppressor cells [5]. Several major immunotherapy modalities have shown rapid progress in recent years, although each continues to face distinct limitations. A comparative summary of these approaches, including recent developments and prospects, is presented in Table 1.

**TABLE 1:** Overview of a few immunotherapy modalities, including mechanisms of action, recent advances and key challenges.

Immunotherapy strategy	Mechanism of action	Recent advances	Challenges/breakthroughs	References
Immune Checkpoint Inhibitors	Block inhibitory immune checkpoint pathways to restore T‐cell activation and enhance antitumor immunity.	PD‐1/PD‐L1 and CTLA‐4 and LAG‐3 inhibitors are used in various cancers, with LAG‐3 inhibitors representing a recent addition to immune checkpoint blockade.	Resistance in some tumors; immune‐related adverse events.	[6, 7]
CAR‐T Cell Therapy	Genetically modified T cells targeting tumor antigens.	Approved for B‐cell malignancies. CAR‐T has been approved for solid tumors in China.	Limited efficacy in solid tumors; risk of CRS and neurotoxicity.	[8]
Cancer Vaccines	Stimulate an immune response to tumor‐specific or neoantigens.	mRNA and neoantigen‐based vaccines are under development.	Need for precise target selection and delivery.	[9]
Oncolytic Virus Therapy	Viruses engineered to kill tumor cells and stimulate immunity.	Examples like T‐VEC are approved; combined with ICIs are being evaluated in trials.	Target specificity, delivery, and immune clearance of viruses.	[10]
Combination Therapy	Uses multiple treatments (e.g., ICIs + chemotherapy/radiation).	Improved outcomes in several cancers.	Optimization of timing, dosage, and patient selection.	[11, 12]
TME Modulation	Altering the TME to favor immune activity.	Strategies to deplete Tregs and MDSCs; promote effector cell function.	Complexity of TME interactions; risk of off‐target effects.	[13]
Next‐Gen CAR‐T Cells	Enhanced CAR constructs for solid tumor targeting.	Dual CARs, switchable CARs, and armored CAR‐Ts in development.	Technical complexity and ensuring safety in heterogeneous tumor settings.	[14]
Precision & AI Integration	Use of genomics and AI to tailor immunotherapy	AI for neoantigen prediction and therapy matching.	Data integration, validation, and clinical translation challenges.	[15]
T‐cell Receptor‐engineered T‐cell therapy	Autologous T cells are genetically engineered to express TCRs that recognize peptide antigens presented by human leukocyte antigen (HLA)/MHC molecules, enabling targeted killing of tumor cells.	Clinical efficacy has been demonstrated in synovial sarcoma and other MAGE‐A4‐positive solid tumors, culminating in the first FDA approval of a TCR‐T therapy in 2024.	Antigen loss and tumor heterogeneity can cause immune escape.	[16, 17]

These features can weaken endogenous antitumor immunity even when tumor antigens are present, highlighting the need for therapeutic strategies capable of actively recruiting and stimulating immune cells within tumors [3, 18, 19].

Among molecules capable of inducing strong immune activation, bacterial superantigens (SAgs) represent a unique class of immunomodulatory proteins produced primarily by pathogens such as *Staphylococcus aureus* and *Streptococcus pyogenes* [20]. Unlike conventional peptide antigens that require intracellular processing and presentation within the peptide‐binding groove of major histocompatibility complex (MHC) molecules, superantigens activate T cells through a distinct mechanism involving the direct crosslinking of MHC class II molecules on antigen‐presenting cells with specific variable β (Vβ) regions of the T‐cell receptor. This unconventional interaction bypasses classical antigen specificity and can activate up to 20%–30% of the total T‐cell population simultaneously, leading to rapid cytokine release and extensive immune activation (Figure 2); [21, 22].

The same biological properties that make superantigens potent immune activators also explain their association with severe inflammatory diseases such as toxic shock syndrome. Systemic exposure to bacterial superantigens can trigger massive cytokine release, leading to widespread immune activation and potentially life‐threatening inflammatory responses. These safety concerns historically limited the therapeutic application of superantigens despite their remarkable capacity to activate polyclonal T‐cell responses [21, 23].

Nevertheless, the ability of superantigens to mobilize large populations of T cells independently of conventional peptide–MHC recognition has generated sustained interest in their potential application for cancer immunotherapy. In principle, superantigen‐mediated T‐cell activation could help overcome a key limitation of many tumors because of the absence of sufficient endogenous antitumor immune responses. By activating broad populations of T cells, superantigens may promote rapid immune infiltration into tumors and facilitate tumor cell destruction. Early experimental studies demonstrated that superantigen exposure could induce strong antitumor immune responses in preclinical models, suggesting that these molecules might serve as powerful immune‐redirecting agents when appropriately controlled [20, 24].

**FIGURE 3:**
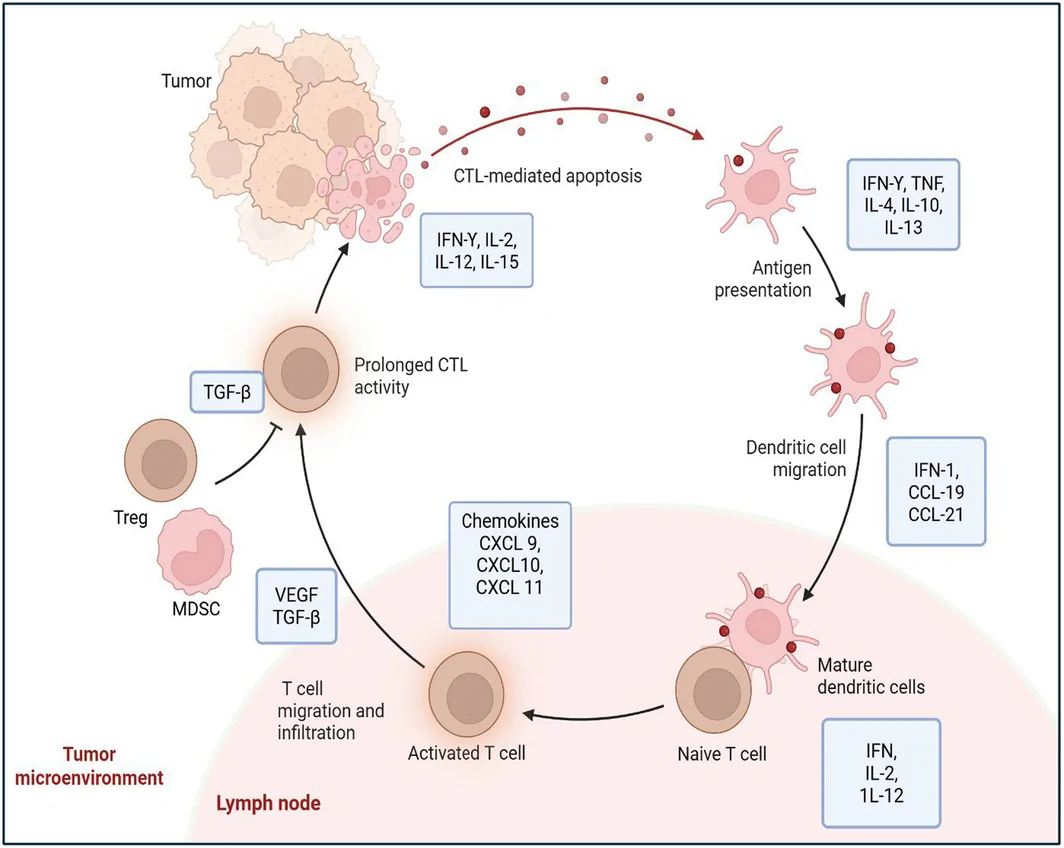
Cytokine Dynamics in the Cancer‐Immunity Cycle. This figure illustrates the complex roles of cytokines throughout the cancer‐immunity cycle, highlighting their contributions to both antitumor responses and immune suppression. Pro‐inflammatory cytokines such as IFN‐γ, IL‐2, IL‐12, and IL‐15 enhance cytotoxic T‐lymphocyte (CTL)‐mediated tumor cell killing within the tumor microenvironment. Conversely, immunosuppressive cells—including myeloid‐derived suppressor cells (MDSCs) and regulatory T cells (Tregs), IL‐10 and TGFβ to dampen CTL activity and promote immune evasion. In lymph nodes, DCs present tumor antigens, a process supported by cytokines such as IFN‐I, IFN‐γ, TNF, IL‐4, IL‐10, and IL‐13. DC migration to lymph nodes is driven by IFN‐I, CCL19, and CCL21, facilitating T‐cell priming. Activated T cells then migrate toward the tumor in response to chemokines including CXCL9, CXCL10, CXCL11, and CCL19. However, factors like VEGF and TGF‐β within the tumor microenvironment can inhibit T‐cell infiltration and function, emphasizing the balance between immune activation and tumor‐mediated suppression.

To overcome the toxicity associated with native superantigens, recent research has focused on engineering modified superantigen constructs that improve tumor selectivity while preserving immune‐activating capacity. Approaches such as attenuation of MHC class II binding affinity and fusion of superantigens to tumor‐targeting antibodies or immune‐engager constructs aim to localize immune activation within tumors while minimizing systemic inflammatory responses [18, 25]. Advances in protein engineering have further expanded the design of multifunctional immune‐engager platforms capable of recruiting multiple immune cell populations, including T cells and natural killer cells, thereby enhancing coordinated cytotoxic responses against tumor cells [26, 27]. Figure 3 explains the mechanism of T cell‐mediated apoptosis in tumor.

The development of engineered superantigen therapeutics has also occurred alongside rapid advances in other T‐cell‐redirecting immunotherapies, including CD3‐based bispecific antibodies and T‐cell receptor (TCR)‐based engagers. These technologies have demonstrated substantial clinical benefit by redirecting cytotoxic immune cells toward tumor‐associated antigens. However, they also face limitations, including dependence on specific antigen presentation, tumor antigen heterogeneity, and immune escape mechanisms that impair antigen recognition. In comparison, superantigens activate T cells independently of peptide antigen recognition, potentially enabling broader immune recruitment even in tumors with defective antigen presentation pathways [24, 28].

Taken together, these developments suggest that superantigen‐based immunotherapy should be reconsidered not simply as a historical concept but as a mechanistically distinct class of immune‐redirecting therapeutics. While their capacity for broad T‐cell activation presents unique safety challenges, advances in molecular engineering, tumor targeting, and immune‐engager design have created new opportunities to harness their immune‐stimulating properties for cancer treatment. Understanding how these strategies improve the therapeutic index of superantigens will be critical for determining their future role in immuno‐oncology [29].

This review provides a comprehensive overview of superantigen‐based strategies for cancer immunotherapy. We first discuss the structural and immunological properties of bacterial superantigens and their mechanisms of T‐cell activation. We then examine engineering approaches developed to improve tumor specificity and reduce systemic toxicity. Subsequently, we analyze current preclinical and clinical studies exploring superantigen‐based immunotherapies, including fusion proteins and targeted delivery platforms. Finally, we discuss emerging combination strategies and future directions for integrating superantigens with modern immunotherapeutic approaches.

## 2 Biological Basis of Superantigens

### 2.1 Structural Features of Superantigens

*Staphylococcal* SAgs are classified as *Staphylococcal enterotoxins* A, B, C (and antigenic variations), D, E, and the newly identified *enterotoxins* G to Q and TSST‐1. While Streptococcal SAgs are classified as pyrogenic exotoxins and include SSA, G‐J, C, A (and antigenic variations) and *Streptococcal mitogenic exotoxin‐Z (SMEZ)*. SAgs derived from bacteria have shown effectiveness in laboratory settings against various cancer cell lines, including those from the liver, colon, stomach, cervix, and breast [30]. In vitro studies typically involve isolating PBMCs from healthy donors. These cells are stimulated with SAgs like *Staphylococcal enterotoxins* (e.g., SEB, SEC1, SEC2), which promote T‐cell proliferation and enhance the production of cytokines, which are key signaling proteins that mediate immune responses. As more PBMCs are stimulated, their ability to inhibit cancer cell growth increases significantly [31]. Various superantigens (SAgs) have been explored for cancer therapy, each differing in their mechanisms of action and key functional characteristics. A comparative overview of selected SAgs is presented in Table 2.

**TABLE 2:** Comparative overview of SAgs in cancer immunotherapy [32, 33].

Superantigen	Mechanism of action	Cytokines released	Targeted cancer	In vitro effectiveness	In vivo effectiveness	Notable findings
SEC1	Stimulates PBMCs, promotes T‐cell proliferation	IL‐2, TNF‐α	Bladder cancer	High T‐cell proliferation, cytotoxicity	Decreases tumor formation, improves survival in mice	Effective in bladder cancer, with strong cytokine release
SEC2	Crosses intestinal epithelium, maintains activity	IL‐2, TNF‐α	Gliomas, intestinal cancer	Moderate T‐cell activation	Enhances chemotherapy/radiotherapy effectiveness	Potential for oral administration, with synergistic effects when combined with other therapies
SAM3	Modified form of SEC2 SAgs	IL‐2, TNF‐α	Various cancer types	High T‐cell activation, lower toxicity	Stronger antitumor activity, lower toxicity	Promising for further development due to lower toxicity
SEB	Induces Th9 cells, stimulates the immune response	IL‐2, TNF‐α	Multiple cancer types	Effective immune activation, tumor inhibition	Induces apoptosis in animal models	Strong immune stimulation, induces Th9 cell response
SEA	Dual binding to MHC class II molecules, T‐cell activation	IL‐2, TNF‐α	Multiple cancer types	Strong T‐cell activation, cytotoxicity	Effective in tumor inhibition across various models	Dual binding site for better T‐cell activation
SED	TCR Vβ‐specific stimulation	IL‐2, IFN‐γ	Not studied	Known potent activator	Not studied	Immunogenic but not yet tested in cancer
SEE	Like SEA/SEB in structure	IL‐2, TNF‐α	Not studied	Likely effective, not tested	Not studied	Shares function with SEA/SEB
TSST‐1	Penetrates mucosa, binds MHC II	IL‐1, IL‐6, TNF‐α	Ovarian (exploratory)	Moderate cytokine induction	Limited tumor studies	Systemic immune activator
SEG	Varying affinities to MHC II and TCR	Variable	Not studied	Unknown	Not studied	Recently discovered, under investigation
SPE‐A	Pyrogenic exotoxin, activates T cells	IL‐2, TNF‐α	Lymphoma (preclinical)	Moderate activation	Tumor shrinkage in early studies	Explored in immuno‐oncology settings
SPE‐C	TCR Vβ‐specific stimulation	IL‐2, TNF‐α	Not studied	Known immune activator	Not studied	Similar to the SEB mechanism
SSA	Superantigenic exotoxin	IL‐2	Not studied	Limited data	Not studied	Mild activity, unexplored in cancer
SMEZ	Broad Vβ‐TCR activation	IL‐2, IFN‐γ	Lymphoid cancers (hypothetical)	Theoretically high	Not studied	Strong in vitro mitogenicity, no cancer models yet

### 2.2 Mechanism of MHC‐TCR Crosslinking

Superantigens activate T cells through a unique mechanism that bypasses conventional antigen processing and presentation. Unlike classical peptide antigens, which are presented within the peptide‐binding groove of major histocompatibility complex (MHC) molecules, superantigens bind simultaneously to the outer surface of MHC class II molecules on antigen‐presenting cells and specific variable regions of the T‐cell receptor β chain (TCR Vβ). This interaction forms a stable MHC‐superantigen‐TCR complex that crosslinks antigen‐presenting cells and T cells independently of antigen specificity. As a result, a large proportion of T cells, often up to 20% of the T‐cell repertoire, can be activated simultaneously, compared with less than 0.01% during conventional antigen recognition. This nonspecific but highly potent activation leads to rapid immune signaling and extensive cytokine release, which underlies both the immunostimulatory potential and the systemic toxicity historically associated with superantigens [20, 21].

### 2.3 Immunological Consequences of Superantigen Activation

The extensive T‐cell activation induced by superantigens results in a profound immunological cascade characterized by the rapid secretion of pro‐inflammatory cytokines such as interleukin‐2 (IL‐2), interferon‐γ (IFN‐γ), and tumor necrosis factor‐α (TNF‐α). This cytokine surge promotes strong immune cell recruitment and amplification of cytotoxic immune responses, which can be therapeutically advantageous in the context of tumor immunity. By activating large populations of T cells irrespective of antigen specificity, superantigens have the capacity to overcome the limited frequency of tumor‐specific T cells that often constrains conventional immunotherapies. However, uncontrolled activation may also lead to systemic inflammatory responses and cytokine release syndrome, which historically limited the clinical application of native superantigens. Modern engineering strategies therefore focus on modifying superantigen structure and delivery to retain their potent immunostimulatory effects while minimizing systemic toxicity [20, 34].

## 3 Engineering Strategies for Superantigen Therapeutics

### 3.1 Antibody‐Superantigen Fusion Constructs

Antibody‐superantigen fusion constructs represent one of the most widely explored strategies for harnessing the potent immune‐activating properties of superantigens while improving tumor specificity. In these engineered molecules, superantigens are genetically fused to monoclonal antibodies or antibody fragments that recognize tumor‐associated antigens [35]. This design enables selective delivery of the superantigen to tumor cells, thereby concentrating T‐cell activation within the tumor microenvironment while reducing systemic toxicity [36]. Upon binding to the tumor antigen, the superantigen component recruits and activates T cells through T‐cell receptor Vβ engagement, resulting in localized immune activation and tumor cell killing. Several constructs based on *staphylococcal enterotoxins* have been developed using this approach, demonstrating enhanced antitumor responses in preclinical models and early clinical trials [23].

### 3.2 Tumor Targeted Superantigen

**FIGURE 4:**
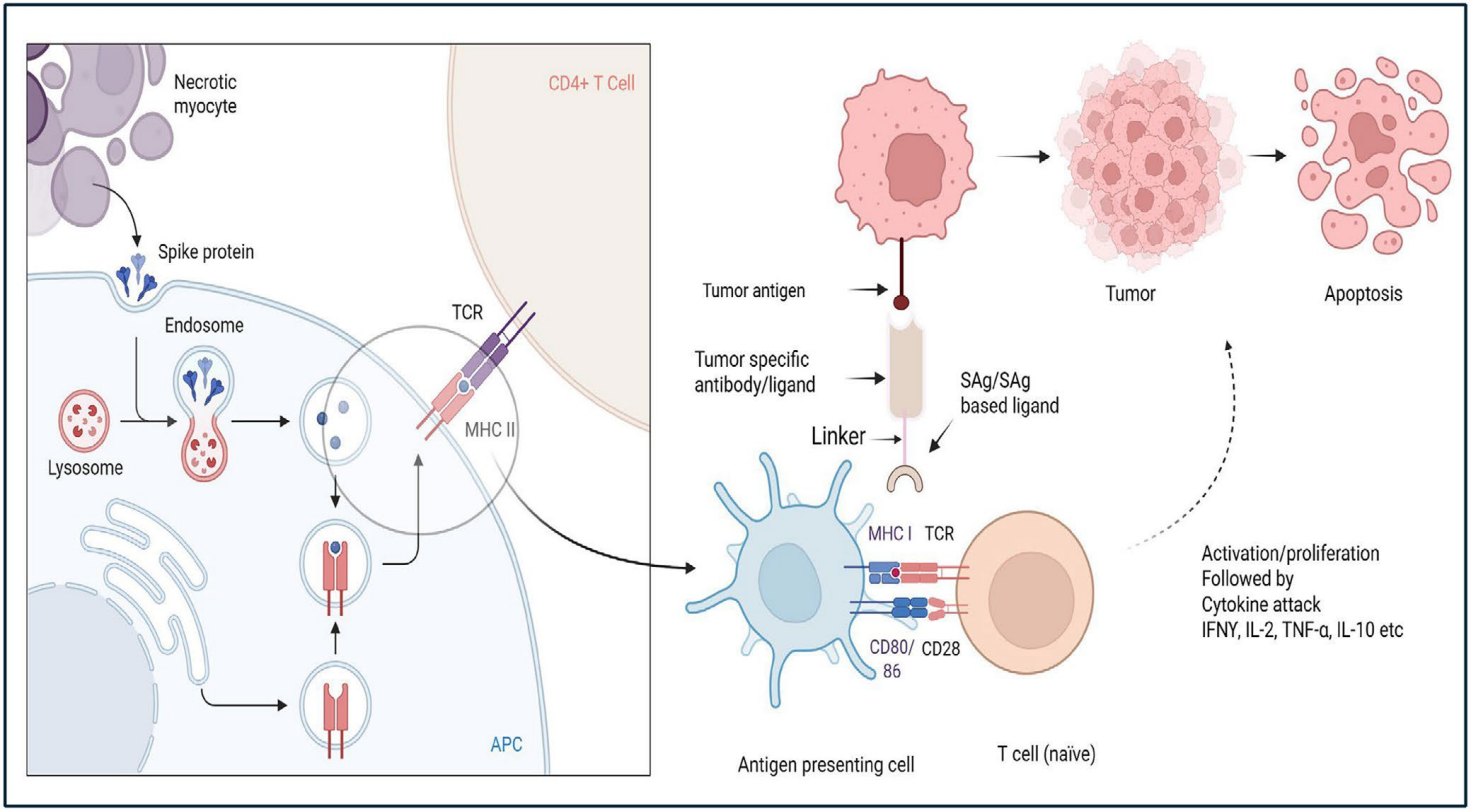
Schematic illustration of a tumor‐targeted superantigen. The tumor‐specific antibody or ligand binds to the tumor antigen, while the SAgs or ligand forms a crosslink between MHC II and TCR, triggering T‐cell activation and cytokine production at the tumor site.

Another major strategy involves engineering superantigens that selectively target tumor cells through receptor‐binding domains or tumor‐associated antigen recognition motifs. By coupling superantigens with tumor‐specific ligands or antibody fragments, these constructs direct T‐cell activation specifically toward malignant tissues. Such tumor‐targeted superantigens have been designed to recognize surface molecules such as EpCAM, HER2, and other tumor‐associated antigens [34]. Once localized to the tumor, the superantigen component induces strong T‐cell activation and cytokine production, thereby amplifying immune‐mediated tumor destruction. This approach aims to exploit the powerful immune‐activating properties of superantigens while minimizing systemic exposure and associated toxicity (Figure 4); [25, 37, 38].

### 3.3 Engineered Superantigen Variants

Advances in protein engineering have enabled the development of modified superantigens with improved safety profiles. Native superantigens possess extremely high affinity for both MHC class II molecules and T‐cell receptors, which contributes to excessive immune activation and cytokine release. To address this limitation, researchers have introduced targeted mutations to alter the binding affinity of superantigens for MHC class II or TCR Vβ regions. These engineered variants retain their ability to activate T cells but exhibit reduced systemic toxicity. Mutational engineering has also been used to enhance tumor selectivity and improve pharmacokinetic properties. Such engineered superantigens represent an important step toward translating superantigen‐based therapies into clinically viable cancer immunotherapies [20, 26].

### 3.4 Delivery Platforms and Novel Engineering Approaches

Beyond protein engineering, emerging delivery strategies are being explored to improve the therapeutic potential of superantigens. Nanoparticle‐based delivery systems, bacterial vectors, and vesicle‐based platforms have been investigated as means of directing superantigen activity toward tumors while minimizing systemic toxicity. These platforms can facilitate controlled release of superantigens within the tumor microenvironment and enhance immune cell recruitment to tumor sites. In addition, advances in synthetic biology and immune‐engager design have enabled the development of multifunctional constructs capable of simultaneously activating multiple immune cell populations. Such innovations highlight the expanding role of engineered superantigen platforms within the broader landscape of cancer immunotherapy [26, 34].

## 4 Tumor Microenvironment Challenges

The tumor microenvironment (TME) plays a critical role in determining the success or failure of cancer immunotherapies. Many solid tumors develop an immunosuppressive microenvironment characterized by low T‐cell infiltration, regulatory immune populations, and inhibitory cytokine signaling. These “cold” tumors are particularly resistant to immune‐based treatments because effective antitumor responses require sufficient numbers of activated cytotoxic T cells within the tumor site. In addition, immunosuppressive cell populations such as regulatory T cells (Tregs) and myeloid‐derived suppressor cells (MDSCs), along with inhibitory checkpoint signaling pathways, can further dampen antitumor immunity and limit therapeutic efficacy [4].

**FIGURE 5:**
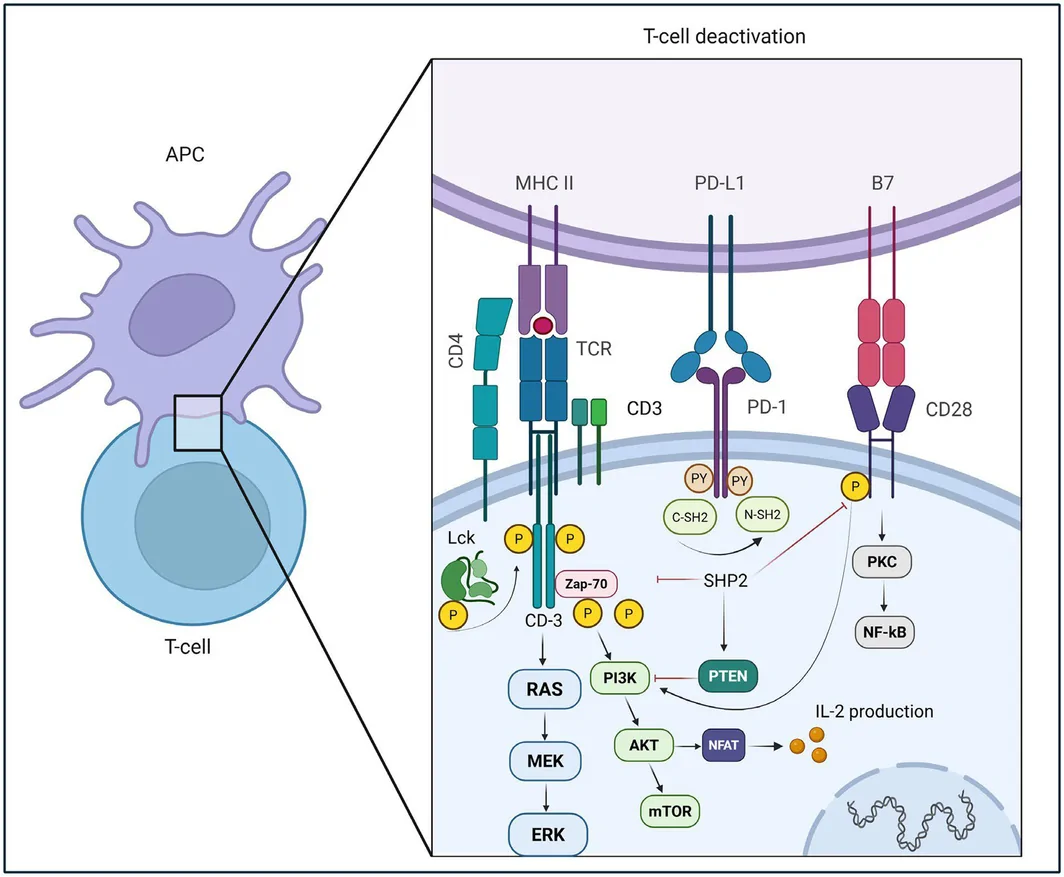
Immune regulatory mechanism of the PD‐1/PD‐L1 axis. Antigen‐presenting cells (APCs) present tumor‐derived antigens to T‐cell receptors (TCRs) through the major histocompatibility complex (MHC). The specific interaction between the MHC‐antigen complex and the TCR initiates intracellular signaling cascades, including the phosphatidylinositol and mitogen‐activated protein kinase (MAPK) pathways, leading to the activation of effector T‐cell immune responses. When PD‐L1 binds to PD‐1, tyrosine residues within the immunoreceptor tyrosine‐based switch motif (ITSM) and immunoreceptor tyrosine‐based inhibitory motif (ITIM) domains of PD‐1 become phosphorylated, resulting in the recruitment and activation of SHP‐2 phosphatase. Activated SHP‐2 then dephosphorylates TCR‐associated CD3 and ZAP70 complexes and suppresses CD28 costimulatory signaling. This collectively dampens TCR signaling and cytokine production, such as IL‐2, ultimately leading to T‐cell inhibition.

Superantigen‐based strategies may offer a potential approach to overcoming these challenges by activating large populations of T cells independently of tumor antigen specificity. Because superantigens engage T‐cell receptors based on Vβ family usage rather than peptide recognition, they can recruit a broad pool of T cells to participate in antitumor responses. When delivered in a tumor‐targeted manner, engineered superantigens may therefore enhance immune cell infiltration and promote local cytokine production within the tumor microenvironment. This property has generated growing interest in combining superantigen‐based therapies with other immunotherapeutic approaches, including immune checkpoint blockade, to improve antitumor efficacy in otherwise immunologically resistant tumors (Figure 5); [26, 34].

## 5 Clinical Development of Superantigen Therapies

Superantigen‐based therapeutics have progressed from early experimental concepts to clinically evaluated immunotherapeutic agents. Initial clinical studies primarily investigated engineered superantigen fusion proteins designed to redirect T‐cell responses toward tumor cells. These constructs typically combine a tumor‐targeting antibody fragment with a modified superantigen, enabling selective recruitment and activation of T cells at the tumor site. By concentrating immune activation within the tumor microenvironment, these approaches aim to retain the potent immunostimulatory capacity of superantigens while minimizing systemic toxicity, as discussed in Table 3.

**TABLE 3:** Clinical development of superantigen therapies ([20, 34]).

Agent	Target/design	Cancer type	Clinical phase	Key findings
Naptumomab estafenatox (ABR‐217620)	Antibody‐superantigen fusion targeting 5T4 antigen	Renal cell carcinoma	Phase II	Demonstrated immune activation and antitumor activity in selected patients
SEA/E‐120	Engineered *staphylococcal enterotoxin*	Various solid tumors	Early clinical development	Modified superantigen designed to reduce toxicity
Superantigen‐based T‐cell engagers	Tumor‐targeted immune activation platforms	Multiple cancers	Preclinical/early clinical	Show potential for strong T‐cell recruitment to tumors

Several engineered superantigen platforms have been evaluated in preclinical and clinical studies for the treatment of different malignancies. Early investigations demonstrated that tumor‐targeted superantigen constructs could induce strong T‐cell activation and cytokine production, resulting in enhanced tumor cell killing. Although challenges related to toxicity and immune regulation remain, advances in protein engineering and targeted delivery strategies have significantly improved the therapeutic potential of these agents. Continued development of superantigen‐based immunotherapies, particularly in combination with other immune‐modulating treatments, may further expand their role within the broader landscape of cancer immunotherapy [20, 34].

## 6 Preclinical Mechanisms of Superantigen‐Mediated Cytotoxicity: SDCC Versus SADCC

Despite their promise, SAgs can also cause severe side effects due to their nonspecific activation of T cells. To address this, various strategies have been developed (a few of them are discussed above). These approaches effectively direct the immune response to tumor sites, leading to the elimination of a large percentage of tumor cells in preclinical models.

Preclinical testing of SAg‐based therapies has revealed two main mechanisms of tumor cell killing: superantigen‐dependent cell‐mediated cytotoxicity (SDCC) and Superantigen antibody‐dependent cell‐mediated cytotoxicity (SADCC).

- SDCC relies on the presence of MHC class II molecules on target cells to activate lymphocytes, primarily CD8+ T cells, leading to cytokine production and cytotoxicity. However, the therapeutic use of SDCC is limited by systemic toxicity and the frequent absence of MHC class II on tumor cells [39].
- To overcome these limitations, SADCC utilizes SAgs fused to monoclonal antibodies targeting tumor‐specific antigens, enabling MHC‐independent tumor targeting. This approach reduces systemic toxicity and enhances tumor‐specific cytotoxicity through localized T‐cell activation and cytokine release. SADCC has demonstrated significant tumor growth inhibition and improved survival in animal models, though it requires repeated administration to maintain efficacy [40]. Preclinical studies have revealed important differences between superantigen‐dependent cellular cytotoxicity (SDCC) and superantigen‐antibody‐dependent cellular cytotoxicity (SADCC) in terms of their mechanisms, target requirements, effector cell engagement, toxicity, and therapeutic efficacy. These differences are summarized in Table 4.

**TABLE 4:** Key mechanistic and immunological differences between SDCC and SADCC [29, 39, 41].

Aspects	Superantigen‐dependent cell‐mediated cytotoxicity	Superantigen antibody‐dependent cell‐mediated cytotoxicity
Mechanism	SAg activates lymphocytes via MHC class II on target cells	SEA fused/conjugated to tumor‐specific mAbs targets tumor cells independently of MHC II
Target cell requirement	MHC class II‐positive only	Targets tumor cells via antibody binding, independent of MHC class II
Cytotoxic effector cells	Mainly CD8+ T cells	CD4+ and CD8+ T cells, requires MHC class II+ monocytes for cytokine mediation
Systemic toxicity	High (toxic shock syndrome due to generalized immune activation)	Reduced due to lower MHC II binding and tumor‐specific targeting
Tumor expression limitation	Limited since many tumors lack MHC class II	Overcomes limitation by antibody targeting tumor antigens
In vitro effect	Cytotoxicity and cytokine production dependent on MHC II	High cytokine production and tumor growth inhibition, even with MHC II‐negative tumors
In vivo efficacy (mouse models)	Activation of CD8+ T cells; IL‐2 receptor expression and proliferation	85%–99% tumor growth inhibition; long‐term survival; requires repeated injections
Cytokines involved	IL‐2, TNF‐α, IFN‐γ (mainly from T cells)	TNF‐α, IL‐2, IFN‐γ (T cells); IL‐4, IL‐10, TNF‐α (tumor cells)
Additional immune effects	None specified	Induces perforin production, activates NK cells/macrophages, upregulates adhesion molecules and MHC II on tumors
Clinical challenges	Systemic toxicity; limited tumor MHC II expression	Requires engineering fusion proteins; repeated dosing needed for sustained effects
Fusion proteins	Not typically utilized.	Employs fusion proteins combining SAgs with tumor‐targeting antibodies (e.g., Naptumomab estafenatox).
Engineered molecules	Limited engineering; relies on natural SAg properties.	Involves engineered bispecific or trispecific molecules to enhance specificity and efficacy.
NK cell engagement	Not a primary mechanism.	Designed to engage NK cells via Fc regions or specific NK cell receptors (e.g., NKp46).
Antibody Engineering	Minimal involvement.	Extensive antibody engineering to improve targeting and reduce immunogenicity.
B Cell Superantigen	Not applicable.	Potential to engage B cell SAgs to modulate immune responses.
T Cell Engagers	Activates T cells broadly via MHC class II interaction.	Utilizes engineered T cell engagers (e.g., ImmTACs) for targeted activation.
Tumor Targeting	Limited to MHC class II^+^ tumors.	Broad targeting through antibody specificity to tumor antigens.
SAg Activation	Direct activation of T cells via MHC class II.	Controlled activation through targeted delivery of SAgs to tumor cells.

## 7 Comparison of Superantigen‐Based Therapies With Other T‐Cell Redirecting Cancer Immunotherapies

T‐cell‐redirecting strategies represent one of the most transformative advances in modern cancer immunotherapy. Several platforms have been developed to harness T cells for tumor eradication, including chimeric antigen receptor (CAR)‐T cells, T‐cell receptor (TCR) engineered therapies, CD3‐bispecific antibodies, and superantigen‐based constructs. While these approaches share the common goal of directing T‐cell cytotoxicity toward malignant cells, they differ substantially in mechanisms of T‐cell engagement, manufacturing complexity, clinical applicability, and safety profiles. Understanding these distinctions is critical for positioning superantigen‐based therapeutics within the evolving immunotherapy landscape.

T‐cell‐redirecting immunotherapies have transformed the treatment landscape of cancer by enabling immune effector cells to recognize and eliminate malignant cells with high specificity. Among these approaches, chimeric antigen receptor (CAR)‐T cells, T‐cell receptor (TCR)‐engineered therapies, and CD3‐bispecific antibodies have demonstrated significant clinical success, particularly in hematologic malignancies. Superantigen‐based therapeutics represent an alternative strategy that exploits the unique ability of superantigens to activate large populations of T cells through Vβ‐specific interactions with the T‐cell receptor (TCR). Understanding how superantigen‐mediated immune activation differs from other T‐cell‐redirecting approaches is essential for positioning these agents within the broader cancer immunotherapy landscape.

### 7.1 Superantigens Versus CAR‐T Cell Therapy

CAR‐T cell therapy involves the genetic modification of patient‐derived T cells to express synthetic receptors that recognize tumor‐associated antigens independently of major histocompatibility complex (MHC) presentation. Following reinfusion, these engineered cells can recognize and kill tumor cells expressing the target antigen with high potency. CAR‐T therapies have achieved remarkable success in hematologic cancers such as B‐cell leukemia and lymphoma, leading to several regulatory approvals [42].

Despite these advances, CAR‐T therapies face significant limitations, including complex manufacturing processes, high treatment costs, and reduced efficacy in solid tumors due to poor tumor infiltration and immunosuppressive tumor microenvironments [43]. In contrast, superantigen‐based therapies do not require ex vivo cell engineering. Instead, they activate endogenous T cells in vivo, enabling rapid recruitment of a large fraction of the T‐cell repertoire. This feature offers the potential for broader immune activation without individualized cell manufacturing [25]. However, the potent immune stimulation associated with superantigens also raises concerns regarding systemic cytokine release and toxicity, necessitating careful engineering of attenuated or tumor‐targeted superantigen constructs.

### 7.2 Superantigens Versus TCR‐Engineered T‐Cell Therapies

TCR‐engineered T‐cell therapies represent another adoptive immunotherapy approach in which T cells are genetically modified to express tumor‐specific T‐cell receptors capable of recognizing peptide antigens presented by MHC molecules. Unlike CAR‐T cells, TCR therapies can target intracellular tumor antigens, expanding the range of potential therapeutic targets [44].

However, these therapies remain constrained by MHC restriction, meaning that treatment efficacy depends on both the patient's HLA type and the presentation of the specific tumor antigen. Furthermore, cross‐reactivity between engineered TCRs and healthy tissue antigens has resulted in severe toxicities in some clinical trials. Superantigens circumvent these limitations by bypassing conventional peptide‐MHC recognition, instead binding directly to the Vβ region of TCRs and crosslinking them with MHC molecules on antigen‐presenting cells. This interaction enables the activation of large polyclonal T‐cell populations, potentially generating stronger immune responses against tumor cells. Nonetheless, this broad activation must be carefully controlled to prevent excessive systemic immune responses.

### 7.3 Superantigens Versus CD3 Bispecific Antibodies

CD3‐bispecific antibodies are another important class of T‐cell‐redirecting therapies that function by simultaneously binding tumor antigens and CD3 molecules on T cells, thereby bringing effector cells into proximity with cancer cells. This interaction promotes targeted cytotoxic activity and has been successfully translated into clinical therapies such as blinatumomab, which is approved for the treatment of B‐cell acute lymphoblastic leukemia [45].

Superantigen‐based therapies share conceptual similarities with bispecific antibodies in that both strategies facilitate the physical interaction between T cells and tumor cells. However, the mode of T‐cell engagement differs substantially. Bispecific antibodies activate T cells through CD3 signaling, whereas superantigens bind directly to the Vβ region of the TCR, allowing the activation of a broader proportion of circulating T cells. This unique mechanism may enable stronger immune amplification but also increases the risk of systemic immune activation if not properly controlled through targeted delivery strategies [46].

### 7.4 Distinct Immunotherapeutic Advantages of Superantigens

Superantigens possess several features that distinguish them from other T‐cell‐redirecting immunotherapies. Their ability to activate a large fraction of the T‐cell repertoire independent of antigen specificity allows rapid immune amplification and potent cytokine production. Additionally, advances in protein engineering have enabled the development of tumor‐targeted superantigen constructs, antibody‐superantigen fusion proteins, and attenuated variants designed to reduce systemic toxicity while preserving antitumor activity [26].

Although CAR‐T cells currently dominate the field of adoptive cell therapy, superantigen‐based strategies offer a complementary approach that may overcome some limitations of existing therapies, particularly in situations where rapid activation of endogenous T cells is advantageous. Continued advances in molecular engineering and targeted delivery may further enhance the therapeutic potential of superantigens within the broader landscape of cancer immunotherapy [21, 24, 28].

## 8 Superantigen‐Based Combination Strategies in Cancer Immunotherapy

While superantigens possess strong intrinsic immunostimulatory properties, their clinical efficacy may be enhanced through combination with other immunotherapeutic strategies that address tumor immune evasion and immunosuppressive microenvironments. Combination approaches have emerged as an important direction in cancer immunotherapy, as they allow multiple mechanisms of antitumor immunity to be engaged simultaneously.

### 8.1 Superantigens and Immune Checkpoint Blockade

Immune checkpoint inhibitors targeting PD‐1, PD‐L1, and CTLA‐4 have transformed cancer treatment by restoring T‐cell activity in tumors that suppress immune responses. However, these therapies rely on the presence of tumor‐reactive T cells within the tumor microenvironment. Because superantigens activate T cells through Vβ‐specific TCR engagement independent of peptide antigen recognition, they may increase the pool of activated T cells capable of responding to checkpoint blockade.

Preclinical work suggests that superantigen‐mediated T‐cell activation can enhance tumor immune infiltration and cytokine production, thereby increasing tumor immunogenicity and improving responses to checkpoint inhibition [47]. In murine tumor models, superantigen‐based constructs have been shown to induce strong T‐cell activation and interferon‐γ production, which can promote antigen presentation and potentially sensitize tumors to checkpoint blockade. These findings support the hypothesis that checkpoint inhibitors may prolong the functional activity of superantigen‐activated T cells, thereby sustaining antitumor responses within immunosuppressive tumor environments.

These findings underscore the importance of targeted superantigen delivery and suggest that patient stratification based on TCR repertoire characteristics may improve clinical outcomes.

Immune checkpoint blockade has revolutionized cancer treatment by targeting inhibitory pathways such as PD‐1/PD‐L1 and CTLA‐4, which suppress T‐cell activity within the tumor microenvironment. Although superantigens can rapidly activate large populations of T cells, these activated cells may become functionally exhausted or inhibited by checkpoint pathways within tumors. Combining superantigen‐based therapies with immune checkpoint inhibitors may therefore enhance sustained T‐cell activity and improve antitumor responses.

**FIGURE 6:**
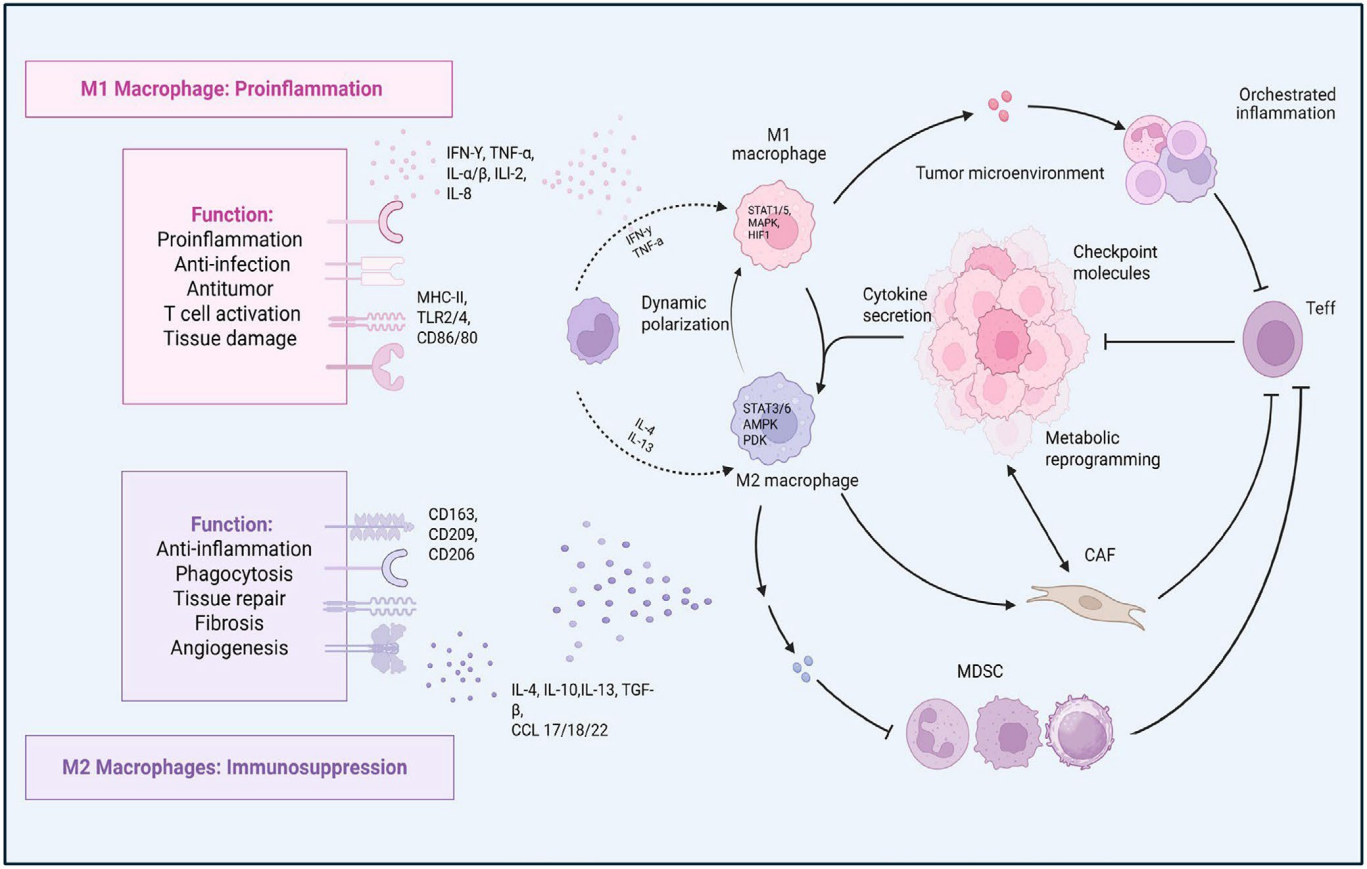
Dynamic polarization and functional roles of M1 and M2 macrophages in the tumor microenvironment. This figure illustrates the dual roles of macrophage polarization in immune regulation. M1 macrophages, activated by stimuli such as IFN‐γ and TNF‐α, exhibit proinflammatory and antitumor functions by producing cytokines including IL‐1α/β, IL‐2, and IL‐8, and expressing surface markers such as MHC‐II, TLR2/4, and CD80/86. These macrophages promote T‐cell activation and tissue damage, contributing to orchestrated inflammation. Conversely, M2 macrophages, induced by IL‐4, IL‐10, IL‐13, and TGF‐β, play an anti‐inflammatory and immunosuppressive role, facilitating tissue repair, fibrosis, and angiogenesis, and express markers such as CD163, CD209, and CD206. Within the tumor microenvironment, macrophages dynamically transition between M1 and M2 phenotypes. M1 macrophages drive proinflammatory responses, while M2 macrophages support tumor progression by secreting cytokines that promote metabolic reprogramming, immune suppression, and activation of cancer‐associated fibroblasts (CAF) and myeloid‐derived suppressor cells (MDSCs). This interplay regulates T‐effector (Teff) cell function and contributes to immune evasion mechanisms in cancer.

Preclinical studies suggest that checkpoint blockade can prolong the functional activity of superantigen‐activated T cells, allowing them to maintain cytotoxic activity against tumor cells despite inhibitory signals present in the tumor microenvironment. Such strategies may be particularly relevant in solid tumors, where immune suppression often limits the efficacy of single‐agent immunotherapies (Figure 6); [48, 49].

### 8.2 Tumor‐Targeted Superantigen Immunotherapies in Clinical Development

One of the most extensively studied strategies for reducing systemic toxicity while maintaining superantigen‐mediated immune activation involves tumor‐targeted antibody‐superantigen fusion proteins. These constructs combine the potent T‐cell‐activating capacity of superantigens with antibody‐mediated tumor targeting.

A prominent example is naptumomab estafenatox (ABR‐217620), a fusion protein consisting of a mutated form of *staphylococcal enterotoxin A* linked to an antibody fragment targeting the tumor antigen 5T4, which is expressed in several solid tumors, including renal cell carcinoma and non‐small‐cell lung cancer. Clinical trials evaluating this construct demonstrated that treatment could selectively activate tumor‐directed T cells and induce measurable immune responses in patients [50]. Importantly, treatment responses were associated with the presence of T‐cell populations expressing specific Vβ subsets capable of responding to the superantigen component, highlighting the importance of T‐cell repertoire composition in determining therapeutic efficacy.

### 8.3 Superantigen‐Based Immune Cell Engagers

Recent advances in protein engineering have enabled the development of next‐generation superantigen‐based immune cell engagers designed to improve tumor targeting while enhancing immune activation. These constructs integrate superantigen scaffolds with tumor‐targeting domains and additional immune‐activating components.

For example, Yu et al. [26] recently reported the development of a high‐affinity superantigen‐based trifunctional immune cell engager capable of simultaneously recruiting both T cells and natural killer (NK) cells to tumor sites. In experimental models, this construct demonstrated enhanced antitumor activity compared with conventional immune cell‐redirecting therapies by promoting coordinated activation of multiple immune effector populations [26]. The ability to recruit both innate and adaptive immune cells represents an important conceptual advance, suggesting that superantigen‐based platforms may serve as versatile scaffolds for multifunctional immunotherapeutic design.

### 8.4 Superantigens as Immune Amplifiers in Multimodal Cancer Immunotherapy

Beyond specific engineered constructs, superantigens may function more broadly as immune amplifiers within multimodal cancer immunotherapy strategies. Their ability to activate large fractions of the T‐cell repertoire enables rapid expansion of effector T‐cell populations capable of mediating tumor cytotoxicity. Experimental studies have demonstrated that superantigens can induce strong cytokine responses and enhance antigen presentation, thereby promoting tumor‐directed immune responses [51].

Advances in molecular engineering have produced attenuated superantigen variants with reduced toxicity while preserving T‐cell‐activating capacity. These engineered molecules may be particularly useful in combination immunotherapy strategies where controlled immune stimulation is required to enhance antitumor immunity without triggering excessive systemic inflammation [34].

Collectively, these findings suggest that the future of superantigen‐based cancer immunotherapy may lie in rationally designed combination strategies that integrate targeted superantigen delivery with complementary immunotherapeutic mechanisms. Continued advances in protein engineering, tumor targeting, and immune modulation are likely to expand the clinical applicability of superantigen‐based therapeutics in cancer treatment.

## 9 Future Directions for Superantigen‐Based Cancer Immunotherapy

Despite their strong immunostimulatory capacity, the clinical translation of superantigen‐based therapies has historically been limited by systemic toxicity and insufficient tumor specificity. Advances in protein engineering, targeted delivery systems, and immunotherapy combinations are now providing new opportunities to overcome these limitations and re‐evaluate superantigens as therapeutic agents in cancer immunotherapy [52].

### 9.1 Engineering Safer Superantigen Variants

One of the major challenges associated with superantigen therapy is cytokine storm and systemic immune activation resulting from extensive T‐cell stimulation. Modern protein engineering approaches have therefore focused on generating mutated or attenuated superantigen variants that retain T‐cell‐activation capacity while reducing excessive inflammatory responses. Mutational modifications in the MHC‐II binding region or TCR interaction domains have been shown to reduce toxicity while maintaining antitumor immune activation [21]. Such engineered variants may improve the therapeutic index of superantigen‐based immunotherapies.

### 9.2 Targeted Delivery of Superantigen Therapeutics

Improving tumor‐specific targeting remains a key priority for the clinical development of superantigen‐based therapies. Antibody‐superantigen fusion proteins represent an important strategy for improving tumor specificity. For example, naptumomab estafenatox, a fusion of mutated *staphylococcal enterotoxin A* with a 5T4‐targeting antibody fragment, has demonstrated tumor‐directed immune activation in clinical studies [50].

### 9.3 Integration With Combination Immunotherapy

Future therapeutic strategies are likely to integrate superantigens with immune checkpoint blockade to enhance sustained antitumor immunity. Because superantigens activate T cells independently of peptide antigen recognition, they may increase immune infiltration in poorly immunogenic tumors and improve responses to checkpoint inhibitors [53]. Combining superantigens with checkpoint inhibitors or other T‐cell‐activating therapies may therefore help sustain antitumor immune responses and overcome tumor‐mediated immune suppression [47].

### 9.4 Biomarker‐Guided Patient Selection

Another emerging research direction involves identifying predictive biomarkers for superantigen responsiveness. The therapeutic activity of superantigens depends on the presence of T cells expressing specific TCR Vβ families, which determines the proportion of T cells capable of responding to the superantigen stimulus. Consequently, profiling the T‐cell receptor repertoire may help identify patient populations most likely to benefit from superantigen‐based immunotherapy [21]. Integrating biomarker‐guided strategies into clinical trials may improve treatment efficacy and patient selection.

## 10 Conclusion

Superantigens represent a unique class of immune modulators capable of activating large populations of T cells independently of conventional antigen processing. Their ability to rapidly amplify immune responses has generated significant interest in their application for cancer immunotherapy. However, the clinical application of superantigens remains constrained by challenges including systemic toxicity, nonspecific immune activation, and limited tumor targeting. Advances in protein engineering and antibody‐directed delivery have partially addressed these issues, but further optimization is required to achieve safe and durable therapeutic responses. With continued advances in molecular engineering and tumor‐targeted delivery strategies, superantigen‐based platforms may emerge as a complementary class of immune‐redirecting therapeutics alongside CAR‐T cells and bispecific antibodies.

## Author Contributions

Urooj Yousaf Virk: conceptualization, study design, literature search, data curation, writing original draft, figure and schematic preparation, and manuscript revision, Hafiza Aasia Malik: literature review support, manuscript refinement, scientific editing, proofreading, and language editing. Muneera Anwer: manuscript refinement, proof reading, language editing, Jennifer Wilson: review and editing, Md Sifat Rahi: visualization, Ming Q. Wei: supervision.

## Funding

The authors have nothing to report.

## Conflicts of Interest

The authors declare no conflicts of interest.

## Data Availability

This article is a review of previously published literature. Data sharing is not applicable to this article as no datasets were generated or analyzed during the current study.
